# Assessment of quality of life in giant ameloblastoma adolescent patients who have had mandible defects reconstructed with a free fibula flap

**DOI:** 10.1186/1477-7819-12-201

**Published:** 2014-07-08

**Authors:** Xiangru Li, Kuicheng Zhu, Fei Liu, Hongwen Li

**Affiliations:** 1 Department of Stomatology, the First Affiliated Hospital of Zhengzhou University, Zhengzhou University, Zhengzhou 450052, Henan, China; 2 Laboratory Animal Center, Zhengzhou University, Zhengzhou 450052, Henan, China; 3 Dermatology, the First Affiliated Hospital of Zhengzhou University, Zhengzhou university, Zhengzhou, Henan 450052, China

**Keywords:** QOL, Free fibula flap, Ameloblastoma, Reconstruction

## Abstract

**Background:**

The reconstruction of mandibular defects after giant ameloblastoma resection is one of the most challenging problems facing reconstructive surgeons. Mandibular resection has been associated with a poor quality of life (QOL), particularly in adolescent patients reconstructed with a free fibula flap. This study aims to evaluate QOL outcomes in adolescent patients who have had mandibular resections of giant ameloblastoma and reconstruction with a free fibula flap and to collect information about their socio-cultural situation.

**Methods:**

The present study assessed 45 adolescent patients who had undergone immediate mandible reconstruction with a free fibula flap for faint ameloblastoma using University of Washington Quality of Life (UW-QOL) and 14-item Oral Health Impact Profile (OHIP-14) questionnaires.

**Results:**

Thirty-five of the 54 questionnaires were returned (64.81%). In the UW-QOL, of the twelve disease-specific domains, the best three scores from the patients related to pain, shoulder and appearance and the worst three scores related to chewing, anxiety and saliva. In the OHIP-14, the lowest-scoring domain was handicap, followed by physical pain and social disability.

**Conclusions:**

Mandibular reconstruction with a free fibular flap significantly influenced the adolescent patients’ QOL. Adolescent patients pay more attention to postoperative facial appearance; this should be considered in surgical planning.

## Background

In the recent histologic classification of odontogenic tumors from the World Health Organization (WHO), ameloblastoma is defined as a benign, locally invasive epithelial odontogenic neoplasm of putative enamel organ origin. Ameloblastomas are benign, locally-invasive odontogenic neoplasms of epithelial origin, which are derived from the tooth-forming apparatus [1]. They account for approximately 60% of odontogenic tumors of the mandible and 10% of all jaw tumors [2]. The tumor was first described by Broca in 1868 and named by Churchill. Although it is considered to be benign, it may show various biological behaviors ranging from cystic expansion to a more aggressive solid mass and even malignant transformation.

Ameloblastomas often present as painless, slow-growing tumors that cause expansion resulting in perforation of the cortical bone and eventually infiltrate into the adjacent soft tissue. Approximately 10 to 15% of all ameloblastoma occur in young patients; it is considered a rare tumor in these groups [3,4].

In recent years, radical resection and immediate reconstruction have become the state-of-the-art procedures in the management of ameloblastoma. Fibula is the free flap most often used to manage extensive ameloblastoma of the mandible because it aims to provide adequate cosmetic and functional results. In 1989, Hidalgo reported a significant series of free fibula flaps for mandibular reconstruction [5]. The free vascularized fibula flap provides the longest segment of bone, with 20 to 30 cm available for harvest. This flap can be used to span an angle-to-angle defect. The bone is also of adequate width and height to allow for placement of osseointegrated dental implants [6,7].

Mandibular reconstruction after resection of a tumor is extremely important as it improves the patient’s quality of life (QOL). Mandibular function and facial aesthetics are aspects that must be preserved [8]. Assessment of the QOL provides information about the psychosocial well-being of patients and the effects of the disease and its treatment. Hence, it is an important tool for evaluating outcome in conjunction with mortality, morbidity, survival and recurrence rates. QOL has become an increasingly important outcome measure for patient’s undergoing treatment for a wide array of illnesses.

The aim of our study was to evaluate by questionnaire the QOL of adolescent patients who have had mandibular defects after trauma or tumor resection and reconstructions with a free fibula flap. To our knowledge, few studies have considered this in relation to adolescent patients’ free fibula flap reconstruction. The findings could potentially provide useful information for physicians and ameloblastoma patients while treatment is being planned.

## Method

### Details of patients

Our study was granted an exemption in writing by the Ethical Review Board of the First Affiliated Hospital of Zhengzhou University. There were 165 cases of ameloblastoma from all age groups diagnosed in the Department of Stomatology at the First Affiliated Hospital of Zhengzhou University from March 2004 to March 2013. Fifty-four patients from this group who were diagnosed by histopathology with giant ameloblastoma, and who underwent immediate mandibular reconstruction with a free fibula flap, were adolescent patients and are included in this study.

They all satisfied the inclusion criteria: the free fibular flap survived completely; there were no severe postoperative complications; they had no previous or synchronous malignancies and no cognitive impairment; and they were at least 12 months after reconstruction and aged less than 24 years.

Fifty-four patients (41 men, 13 women) met the inclusion criteria. Most adolescent patients completed the questionnaire when they returned to the hospital for review. The remaining patients received a formal letter explaining the study, an informed consent form, and the University of Washington Quality of Life (UW-QOL) and the 14-item Oral Health Impact Profile (OHIP-14) questionnaires, as well as a questionnaire exploring social and educational status. Patients who did not reply within one month received a reminder. Patients, who could not fill in the questionnaires themselves, for example because of dementia or language problems, were excluded from the study.

We compiled and reviewed data for preoperative variables (tumor primary site, sex, age); reconstruction flaps characteristics (flap complications and flap success); follow-up information (recurrence and survival situation).

#### Questionnaires and data collection

Although many generic QOL instruments have been developed over the past 30 years, the OHIP-14 [9] and the UW-QOL [10] are most commonly used for cancer patients. The UW-QOL scale is filled in by the patient and provides a broad measure of QOL for patients with head and neck cancer with good acceptability, practicality, validity, reliability, and responsiveness [11]. The questionnaire is composed of 15 domains: 12 are disease-specific items (pain, appearance, activity, recreation, swallowing, chewing, speech, shoulder, taste, saliva, mood, and anxiety), and 3 are global questions. Each of the twelve included questions has three to six response options. The domains are scored on a scale ranging from 0 (worst) to 100 (best). Besides the 15 questions, patients were asked to choose no more than 3 of the 12 disease-specific domains that had been the most important to them in the preceding 7 days. We scored the individual domains according to the UW-QOL guidelines. The standard UW-QOL is available as a Chinese version and has been validated for a Chinese population [12].

The OHIP-14 consists of 14 items divided into 7 different domains: functional limitation, physical pain, psychological discomfort, physical disability, psychological disability, social disability, and handicap. Each item is scored as: 0 = never; 1 = hardly ever; 2 = sometimes; 3 = fairly often; and 4 = very often. The domains are scored on a scale ranging from 0 (best) to 100 (worst). The higher the score, the poorer the patient’s state of health. The standard OHIP-14 is available as a Chinese version and has been validated for a Chinese population [12].

### Statistical analysis

Data were recorded and analyzed with SPSS 16.0 statistical software (SPSS Inc., Chicago, IL, USA). The significance level was set at *P* < 0.05.

## Result

### Patients

Of the 54 patients who were sent a questionnaire, 35 (64.81%) completed and returned it. Eight questionnaires were returned stating that the patients had changed their address, six patients declined to participate, and five claimed not to have received the questionnaire or the reminder. Of the 35 patients who completed the questionnaires there were 23 men and 12 women, median age 17 (range 10 to 24). The postoperative follow-up period ranged from 12 to 32 months. Details of the patients and their tumors are shown in Table 1.

**Table 1:** Patient profiles

**Variables**	**N**	**%**
Age		
< 18 years	26	74.29%
18 to 24 years	9	25.71%
Gender		
Male	23	65.71%
Female	12	34.29%
Primary tumor sites		
Body-angle	15	42.86%
Body-angle-ramus	12	34.28%
Body	8	22.86%
Clinical type		
Solid	26	74.29%
Unicystic	9	25.71%
Radiographic appearance		
Unilocular	24	68.57%
Multilocular	11	31.43%

There were 14 (40.0%) tumors on the left side, 12 (34.28%) on the right, and 9 (25.72%) that crossed the midline. There were 15 (42.86%) tumors in the body and angle of the mandible, 12 (34.28%) that occupied the body-angle-ramus of the mandible, 8 (22.86%) in the body of the mandible. Sizes of the lesions ranged from 3.8 × 1.3 cm^2^ to 6.4 × 4.3 cm^2^. Radiographic appearance in 24 (68.57%) cases was unilocular; 11(31.43%) cases were multilocular. Clinical classification included 26 (74.29%) solid type and 9 (25.71%) unicystic type tumors.

### Quality of life

UW-QOL: the scores for 12 disease-specific domains and the importance of each domain are all shown in Table 2. Topping the list of GOOD-scoring domains was pain (82.21 ± 5.78), 42.85% of patients scoring ≥ 80% in pain domain. Of the twelve disease-specific domains, the best three scores were for pain, shoulder and appearance; the worst three scores were for chewing, anxiety and saliva. Chewing was considered to be the most important issue in the previous seven days, followed by speech and appearance.

**Table 2:** Means of scores of items and scales of UW-QOL questionnaire

	**Mean**	**SD**	**Median**	**Range**	*GOOD score*, %*	Importance of domains, %*	**Rank order**
UW-QOL							
Pain	82.21	5.78	82.00	60 to 93	*42.85%*	0%	11
Appearance	78.12	11.56	78.00	60 to 90	*25.71%*	*48.57%*	3
Activity	69.48	7.56	69.00	40 to 84	*8.57%*	17.14%	7
Recreation	68.21	10.59	68.00	50 to 78	*0%*	14.29%	8
Swallowing	77.32	6.77	77.00	45 to 88	*28.57%*	*5.71%*	10
Chewing	28.48	3.18	29.00	0 to 68	0%	*77.14%*	1
Speech	71.26	12.57	71.00	35 to 87	*22.86%*	*54.29%*	2
Shoulder	80.29	9.01	81.00	55 to 88	*40.0%*	0%	11
Taste	71.23	8.76	71.00	52 to 88	*25.71%*	*11.43%*	9
Saliva	60.02	7.62	60.00	30 to 82	*42.42%*	*22.86%*	5
Mood	67.09	1.15	68.00	30 to 90	*11.42%*	*20.00%*	6
Anxiety	55.76	8.23	55.00	26 to 70	0%	28.57%	4

*GOOD score %:% scoring ≥ 80.

Importance of domains: percentage of patients that considered that particular domain important (up to three domains per patient).

OHIP-14: distributions of OHIP-14 domain scores at presentation are shown in Table 3. The best domain scores for the complete group were 36.34 for handicap, 38.54 for physical pain, and 40.24 for social disability. The highest score was for psychological disability (53.45 ± 2.54) and physical disability (73.14 ± 10.24). No patients scored 40% or less in physical disability.

There were a total of twenty-six (74.29%) adolescent patients in school. Four (11.42%) patients in primary education. Seven patients (20.00%) in junior middle school, nine (25.71%) patients in senior school, six patients (17.14%) in university graduation. Four patients could not read or write, and needed parents help to complete the questionnaire.

## Discussion

Ameloblastomas are benign, locally-invasive odontogenic neoplasms of epithelial origin, which are derived from the tooth-forming apparatus. As the name implies, it was assumed that ameloblastomas are derived from ameloblasts. This assumption has now almost certainly been confirmed in studies that have shown that the cells of origin are epithelial cells that can express amelogenin [13]. The treatment of giant ameloblastoma tumors affecting the jaw bones often requires extensive resection and results in loss of bone continuity, modifying the contour of the face. Mandibular resection often involves the loss of an important bone segment, including teeth. Mandibular bone defects can cause asymmetry, facial disharmony, and tooth loss which compromises chewing. The mandible plays a major role in airway protection and support of the tongue, lower dentition, and the muscles of the floor of the mouth permitting mastication, articulation, deglutition, and respiration. It also defines the contour of the lower third of the face. Interruption of mandibular continuity, therefore, produces both a cosmetic and functional deformity [14].

The free fibula flap as a source of vascularized bone in reconstructive surgery is in wide use. The fibula has been demonstrated to be an ideal flap for mandibular reconstruction. This is particularly true when a limited number of fibular osteotomies are needed to provide appropriate bone shape. When the needs of reconstruction involve muscle bulk, the flexor hallucis longus can be incorporated in the flap. Because the craniofacial bones, teeth, and soft tissues of adolescent are not completely developed, surgery has the potential to cause irreversible deformities, presenting a great challenge for doctors, who must not only treat the disease, but also consider the aesthetic results in light of the postoperative growth and development of these adolescent patients.

The expectation of clinical outcome of reconstruction is regarded to be the most important factor in the decision, and QOL measurement provides information about perceptions of patients [15]. The relatively large number of questionnaires specific for diseases of the oral cavity reflects the fact that there is no ‘gold standard’. We used the OHIP-14 and UW-QOL questionnaires to assess the postoperative QOL of adolescent patients and the possible relation to reconstructive surgery with a free fibula flap.

The oral specific questionnaire was able to better demonstrate the changes in quality of life due to surgery. Many scholars have chosen to use the UW-QOL questionnaire. The UW-QOL measure was chosen as the head and neck specific questionnaire because it is short and easy for patients to complete themselves, thus making it ideal in a busy outpatient setting. We can see that the highest score of UW-QOL subscales in this study was in the pain domain (82.21 ± 5.78), 42.85% of patients scoring ≥ 80% in this domain, which indicated a slight damage in the pain domain. Of the twelve disease-specific domains, the best three scores were for pain, shoulder and appearance; the worst three scores were for chewing, anxiety and mood. A remarkable finding was that the high score of UW-QOL subscales in this study was in the pain domain. The mean score was 82.21, which indicated a great improvement in pain. We found anxiety (55.76 ± 8.23) and mood (67.09 ± 1.15) domains to have a low score. This suggested that some adolescent patients were in a bad psychological state and that the surgery had some negative influence.

The appearance domain of UW-QOL had been identified as a predictor of the overall burden of oral cancer patients. We found that 48.57% patients were satisfied with the appearance domain. This may be due to free fibula flaps providing us with the opportunity to more carefully address the aesthetic and functional reconstruction of mandible defects based on the wide variety of bone and soft tissue available. The reconstruction of the mandible retained the contour of the lower third of the face, thus, giving patients a better postoperative facial appearance.

However, a remarkable finding was that the lowest score of UW-QOL was in the domain of chewing. This may be because mandibular defects cause the loss of some teeth and, therefore, the ability to chew. This confirms the report of Rogers *et al*. [16] who showed that patients had chewing problems after segmental mandibulectomy and reconstruction using a composite free tissue transfer. Dental implants can resolve this problem, but are difficult in Chinese patients because immediate dental implant placement prolongs rehabilitation time, increases the number of required surgical procedures and imposes an additional economic burden. However, we believe that dental implant placement should be considered an integral part of treatment, especially in adolescent patients.

Hsing *et al*. and Rogers *et al*., in their studies on importance-rating using the UW-QOL questionnaire in patients treated by primary surgery for oral cancer, found that they tended to rate speech, chewing, and swallowing as more important than the other UW-QOL domains. Other studies of importance-rating include Lin *et al*. whose results emphasised swallowing, chewing and speech as important and Li *et al*. whose results rated chewing, swallowing, and saliva [11,12,14] as important. Wang *et al*. in their study on importance-rating used the UW-QOL in similar patients to ours and found that patients tended to rate swallowing, chewing, and speech as more important than the other UW-QOL domains [15]. We found that chewing, speech, and appearance were the most important. This is maybe because in traditional Chinese culture we pay more attention to diet than to other domains.

The OHIP-14 was designed to provide a comprehensive measure of the dysfunction, discomfort, and disability attributed to oral conditions. The OHIP-14 consists of 14 items organized into 7 subscales that assess how oral health can affect physical and social wellbeing. In addition the patient can complete it in ten minutes. There were some lower score in the domains of Handicap (36.34 ± 0.95), Physical Pain (38.54 ± 1.11) and Social disability (40.24 ± 10.24); however, postoperatively there were higher scores for (73.14 ± 10.24) and Physical Disability (53.45 ± 754). No patients scored 40% or less in Physical Disability, which shows that operations for mandibular resections of giant ameloblastoma in adolescent patients and reconstruction with a free fibula flap do have an overall effect on oral health.

Twenty-six (74.29%) adolescent patients in school. Four (11.42%) patients in primary education. Seven patients (20.00%) in junior middle school, nine (25.71%) patients in senior school, six patients (17.14%) in university graduation. Four patients could not read or write, and needed parents help to complete the questionnaire. For some adolescent patients, physical and psychological immaturity made illness interpretation very difficult for doctors and so parents needed to assist by giving the correct interpretation of their child’s illness. The UW-QOL and OHIP-14 do not contain a section on the effect of the free fibula flap donor site on QOL and function. The free fibula flap’s primary donor site left a scar, but as the donor site closure is hidden, patients can easily accept donor site morbidity. In our study, some patients were worried about moving the operated limb, thereby reducing the normal activities. However, Farhadi *et al*. found no clinically obvious difference in movement between the donor and un-operated limbs [15]. We compared the patients’ donor and un-operated control legs with regard to range of motion of ankle inversion and eversion and normal leg movements. All patients showed that there were no significant differences between the two leg movements after 12 months.

The radiographic appearance of ameloblastoma in adolescents can be classified as unilocular or multilocular. In studies by Kahn *et al*. [17] and Huang *et al*. [18], the majority of tumors were unilocular (68.42% and 66.67%, respectively). This was also true of the present study, in which 68.57% (24/35) of the patients had unilocular lesions. Most authors have reported that [19] unilocular tumors have more complete cystic walls with weak local infiltration, a small degree of bone involvement, and no residual tumor after thorough curettage. However, multilocular tumors are highly destructive and invasive, and conservative treatment often leaves residual tumor, frequently recurrent.

Because the craniofacial bones, teeth, and soft tissues of adolescents are not completely developed, surgery has the potential to cause irreversible deformities, presenting a great challenge for doctors, who must not only to treat the disease, but also consider the aesthetic results in light of the postoperative growth and development of these adolescent patients.

There were some limitations in our study. First, this was not a randomized study. Selection bias inevitably existed. Second, although the treatment guidelines are standardized at the study institute, individual variations among surgeons certainly exist. Finally, the time from treatment to questionnaire was not uniform for each patient.

## Conclusions

Immediate mandibular reconstruction with a free fibula flap for giant ameloblastoma patients would have significantly influenced the adolescent patients’ quality of life, especially regarding their chewing, anxiety levels and mental health. Adolescent patients pay more attention to appearance and activity domains than other age groups, so this should be considered for surgical planning.

## Competing interests

The authors declare that they have no competing interests.

## Authors’ contributions

XL, KZ and FL participated in the clinical management of the patient and wrote the manuscript. HL were involved in the final editing. All authors read and approved the final manuscript.

## References

[B1] Snead ML Luo W Hsu DD Melrose RJ Lau EC Stenman G Human ameloblastoma tumors express the amelogenin gene Oral Surg Oral Med Oral Pathol 1992 74 64 72 1508512 10.1016/0030-4220(92)90217-e

[B2] Olaitan AA Adekeye EO Clinical features and management of ameloblastoma of the mandible in children and adolescents Br J Oral Maxillofac Surg 1996 34 248 251 8818260 10.1016/s0266-4356(96)90279-x

[B3] Ghandhi D Ayoub AF Pogrel MA MacDonald G Brocklebank LM Moos KF Ameloblastoma: a surgeon’s dilemma J Oral Maxillofac Surg 2006 64 1010 1014 16781332 10.1016/j.joms.2006.03.022

[B4] Li W Liu F Zhongfei X Huang S Zhu W Sun C Treatment of ameloblastoma in children and adolescents J Hard Tissue Biol 2012 21 121 126

[B5] Hidalgo DA Fibula free-flap: a new method of mandible reconstruction Plast Reconstr Surg 1989 84 71 79 2734406

[B6] Cordeiro PG Disa JJ Hidalgo DA Hu QY Reconstruction of the mandible with osseous free flaps: a 10-year experience with 150 consecutive patients Plast Reconstr Surg 1999 104 1314 1320 10513911 10.1097/00006534-199910000-00011

[B7] Chang YM Wallace CG Tsai CY Shen YF Hsu YM Wei FC Dental implant outcome after primary implantation into double-barreled fibula osteoseptocutaneous free flap-reconstructed mandible Plast Reconstr Surg 2011 128 1220 1228 22094740 10.1097/PRS.0b013e318230c6a9

[B8] Murphy BA Ridner S Wells N Dietrich M Quality of life research in head and neck cancer: a review of the current state of the science Crit Rev Oncol Hematol 2007 62 251 267 17408963 10.1016/j.critrevonc.2006.07.005

[B9] Slade GD Spencer AJ Development and evaluation of the Oral Health Impact Profile Community Dent Health 1994 11 3 11 8193981

[B10] Rogers SN Lowe D Fisher SE Brown JS Vaughan ED Health-related quality of life and clinical function after primary surgery for oral cancer Br J Oral Maxillofac Surg 2002 40 11 18 11883963 10.1054/bjom.2001.0706

[B11] Rogers SN Gwane S Lowe D Humphris G Yueh B Weymuller EA The addition of mood and anxiety domains to the University of Washington quality of life scale Head Neck 2002 24 521 529 12112548 10.1002/hed.10106

[B12] Li W Yang Y Xu Z Liu F Cheng Y Xu L Changfu S Assessment of quality of life of patients with oral cavity cancer who have had defects reconstructed with free anterolateral thigh perforator flaps Br J Oral Maxillofac Surg 2013 51 497 501 23107622 10.1016/j.bjoms.2012.09.005

[B13] Kumamoto H Yoshida M Ooya K Immunohistochemical detection of amelogenin and cytokeratin 19 in epithelial odontogenic tumors Oral Dis 2001 7 171 176 11495193

[B14] Wang L Su YX Liao GQ Quality of life in osteoradionecrosis patients after mandible primary reconstruction with free fibula flap Oral Surg Oral Med Oral Pathol Oral Radiol Endod 2009 108 162 168 19464214 10.1016/j.tripleo.2009.03.005

[B15] Farhadi J Valderrabano V Kunz C Kern R Hinterman B Pierer G Free fibula donor-site morbidity: clinical and biomechanical analysis Ann Plast Surg 2007 58 405 410 17413883 10.1097/01.sap.0000241948.36784.4e

[B16] Rogers SN Devine J Lowe D Shokar P Brown JS Vaugman ED Longitudinal health-related quality of life after mandibular resection for oral cancer: a comparison between rim and segment Head Neck 2004 26 54 62 14724907 10.1002/hed.10351

[B17] Huang IY Lai ST Chen CH Chen CM Wu CW Shen YH Surgical management of ameloblastoma in children Oral Surg Oral Med Oral Pathol Oral Radiol Endod 2007 104 478 485 17524681 10.1016/j.tripleo.2007.01.033

[B18] Kahn MA Ameloblastoma in young persons: a clinicopathologic analysis and etiologic investigation Oral Surg Oral Med Oral Pathol 1989 67 706 715 2544844 10.1016/0030-4220(89)90013-3

[B19] Altini M Coleman H Kieser J Kola H Sneider P Three-dimensional computed tomography reconstruction in treatment planning for large ameloblastoma Ora Surg Oral Med Oral Pathol Oral Radiol Endod 1996 81 619 622 10.1016/s1079-2104(96)80059-x 8734714

